# Effects of outcome revaluation on attentional prioritisation of reward-related stimuli

**DOI:** 10.1177/17470218241236711

**Published:** 2024-03-15

**Authors:** Jenny T Le, Poppy Watson, Mike E Le Pelley

**Affiliations:** 1 School of Psychology, UNSW Sydney, Sydney, NSW, Australia; 2 University of Technology, Sydney, NSW, Australia

**Keywords:** Attention capture, reward, sign-tracking, devaluation

## Abstract

Stimuli associated with rewards can acquire the ability to capture our attention independently of our goals and intentions. Here, we examined whether attentional prioritisation of reward-related cues is sensitive to changes in the value of the reward itself. To this end, we incorporated an instructed outcome devaluation (Experiment 1a), “super-valuation” (Experiment 1b), or value switch (Experiment 2) into a visual search task, using eye-tracking to examine attentional prioritisation of stimuli signalling high- and low-value rewards. In Experiments 1a and 1b, we found that prioritisation of high- and low-value stimuli was insensitive to devaluation of a previously high-value outcome, and super-valuation of a previously low-value outcome, even when participants were provided with further experience of receiving that outcome. In Experiment 2, following a value-switch manipulation, we found that prioritisation of a high-value stimulus could not be overcome with knowledge of the new values of outcomes alone. Only when provided with further experience of receiving the outcomes did patterns of attentional prioritisation of high- and low-value stimuli switch, in line with the updated values of the outcomes they signalled. To reconcile these findings, we suggest that participants were motivated to engage in effortful updating of attentional control settings when there was a relative difference between reward values at test (Experiment 2) but that previous settings were allowed to persist when both outcomes had the same value at test (Experiments 1a and 1b). These findings provide a novel framework to further understand the role of cognitive control in driving reward-modulated attention and behaviour.

## Introduction

Our attention often operates in a goal-directed way, prioritising stimuli that are relevant to our current intentions (Yantis, 2000). But attention can also operate independently of our goals: for example, research shows that prior experience with rewards can influence whether stimuli will automatically capture attention (see Failing & Theeuwes, 2018; Rusz et al., 2020). This effect of reward on attention is notable because the modern world is filled with reward cues: wrappers on high-calorie foods, billboards showing attractive models, advertisements for alcohol and cigarettes, and the bright flashing lights of gambling machines. Through repeated pairings with rewards (pleasurable “highs,” feelings of satiation, monetary wins), these reward cues may become “motivational magnets” that have the power to elicit approach behaviours (Berridge & Robinson, 1998).

Many procedures have been developed to study the influence of reward learning on attentional priority (for reviews, see Failing & Theeuwes, 2018; Rusz et al., 2020). Here we focus on a procedure used by Pearson et al. (2016), which formed the basis of the current study. On each trial, participants are presented with an array of shapes: one diamond (the *target*) and several circles. Participants must make a rapid eye-movement (saccade) to the diamond target to earn a reward. The array also features one coloured *distractor* circle (orange or blue; all other shapes are grey), with the distractor’s colour signalling whether high or low reward is available for a correct response. Importantly, while the distractor signals reward magnitude, participants are told that the reward will be omitted if they look at the coloured distractor. Hence, looking at distractors is counterproductive to participants’ goal of earning points. Yet many studies have found that this exactly is what they do (e.g., Le Pelley et al., 2015; Pearson et al., 2016; Watson, Pearson, Chow, et al., 2019). Notably, participants’ attention is more likely to be captured by the distractor signalling a high-value reward, even though this results in cancellation of a larger reward (relative to looking at the distractor that signals low-value reward). This bias towards the high-reward distractor is most pronounced among the fastest saccades that participants make (~200 ms after onset of the search display: Pearson et al., 2016). The bias also persists—for a while at least—into a subsequent unrewarded phase in which participants are explicitly told that rewards are no longer available, such that distractor colours no longer provide useful information regarding reward availability (Watson, Pearson, Most, et al., 2019). The implication is that capture is not driven by the current informational value of distractors, but is instead a consequence of participants’ prior experience of reward (see also Le Pelley et al., 2017; Pearson et al., in press). Taken together, these findings suggest that the attentional system rapidly prioritises stimuli associated with high-value reward, even when doing so is counterproductive, and when the rewards are no longer delivered. This effect has been termed *value-modulated attentional capture* (Anderson et al., 2011; Pearson et al., 2016).

The findings outlined above demonstrate that reward-associated stimuli can capture attention independently of goals. In this regard we can conceptualise attentional prioritisation as an automatic “response” that can be conditioned through learned associations with reward. The current study probed further the conditions under which stimuli elicit conditioned attentional responses. We investigated whether reward-driven prioritisation is mediated by retrieval of the current value of the outcome signalled by a stimulus—and hence updates flexibly in response to changes in outcome value—or whether prioritisation can become divorced from the value of the events involved, persisting despite changes in outcome value. Answering this question would provide insight into the adaptive nature of attentional control in response to a changing environment (Anderson, 2021). Beyond this theoretical contribution, it has been argued that inflexible reward-related attentional biases play a role in compulsive behaviours and substance use (e.g., Albertella et al., 2020; Albertella, Le Pelley, Chamberlain, et al., 2019; Colaizzi et al., 2020); hence, clarifying conditions that promote or discourage flexibility may shed light on why maladaptive behaviours are maintained and how they may be treated.

The question of whether reward-driven prioritisation is mediated by a representation of the outcome—and hence sensitive to post-conditioning changes in the value of that outcome—has been a target of prior research (De Tommaso et al., 2017; De Tommaso & Turatto, 2021; Pool et al., 2014). In these studies, participants initially learned that one image signalled a high probability of delivery of a desirable chocolate odour (Pool et al., 2014) or drink reward (De Tommaso et al.; De Tommaso & Turatto), whereas another image signalled a low (or zero) probability of the odour/drink. In a subsequent (unrewarded) search task, participants were faster to locate and respond to a target when it appeared in the same location as the high-reward cue than the low-reward cue, indicating a reward-related attentional bias. Participants then ate or drank to satiety, rendering the outcome associated with the cues less desirable. The key question was whether this change in value would diminish the bias to the high-reward cue as measured in a repeat of the search task. Findings were mixed: Pool et al. found some evidence consistent with a reduction—suggesting a flexible bias mediated by a representation of outcome value—whereas data from De Tommaso and colleagues were more consistent with an inflexible bias that was unaffected by a reduction in outcome value.

These prior studies have limitations, however. First, in the task used to assess attention, the target location was independent of cue image location—and consequently there was no specific cost to participants for prioritising attention to the high-reward cue. Here we assume that a participant must attend to a given location to determine if the target is at that location (a fundamental premise of visual search). So in effect the participant must choose an order in which to search the potential target locations. As target location was independent of the location of the reward-signalling cues, any strategy for choosing this search order would be just as good (and just as bad) as any other. For example, with two potential target locations (e.g., De Tommaso & Turatto, 2021; Pool et al., 2014), a strategy of prioritising the high-reward cue would result in a first shift of attention to the correct (target) location on half of trials—as would any other strategy. So there was no particular penalty to this “prioritise the high-reward cue” strategy, and since the high-reward cue was presumably a preferred stimulus (due to its prior association with desirable reward), it seems plausible that participants would have adopted this strategy—even if they had been told that these cues were irrelevant to the location of the target. Consequently, under these conditions any attentional bias to the high-reward cue may reflect strategic, top–down prioritisation of prior signals of reward, rather than an automatic, reflexive, conditioned effect. Hence we cannot know the level at which any effect (or lack of effect) of devaluation is mediated, and this may explain the mixed findings: if procedural differences influenced the balance between strategic and automatic processes. A second limitation is that these studies examined only the influence of a *decrease* in outcome value on attention—it remains possible that an increase in outcome value may have a different effect (and may provide greater motivation to update established behaviour patterns).

Other research has examined the flexibility of reward-related attention via tasks assessing value-modulated attentional capture, conceptually similar to the Pearson et al. (2016) procedure described above (Albertella, Watson, Yücel, et al., 2019; Liao & Anderson, 2020). During this test of attention there is always a specific cost in attending to the reward-signalling distractor. This is because in this procedure the target *never* appears in the location of the reward-signalling distractor item, such that attending to this distractor is demonstrably a worse strategy than any other. Consequently, this approach provides a more diagnostic index of automatic patterns of reward-conditioned attentional capture. In these prior studies, the critical stimuli signalled either high- or low-value reward in an initial phase, before these relationships were switched in a “reversal” phase (i.e., the stimulus that previously signalled high-value reward now signalled low-value reward, and vice versa). Patterns of attention changed in line with the new relationships, demonstrating that conditioned attention can remain flexible to changes in reward structure. However, these studies again fall short of demonstrating that reward-modulated attentional capture is mediated by a representation of the current value of the outcome. This is because these studies changed the *identity* of the outcome paired with each stimulus, rather than just the *value* of that outcome (see also De Tommaso & Turatto, 2021, Experiment 3). During the reversal phase, participants received experience of each stimulus being paired with a new outcome (e.g., a high-reward value) and so any change in attention during this phase may result from new conditioning based on this experience, rather than reflecting mediation of the *association formed during initial training* by knowledge of outcome value. That is, these studies are targeted at investigating the process of reversal learning rather than the outcome (in)dependence of the resulting associations (cf. Panayi & Killcross, 2018).

**Figure 1.**
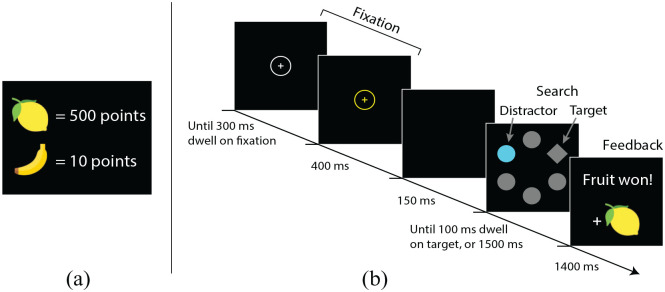
The attentional revaluation task. (a) Participants were told the values of each fruit at the outset, and reminded before each block of trials. (b) On each trial, the colour of a colour-singleton distractor circle in the search display signalled the type of fruit available for making a rapid saccade to the diamond-shaped target. The example here shows a high-training distractor trial, where a blue distractor signals availability of a lemon worth 500 points (fruit-value and colour-fruit contingencies were counterbalanced across participants). If participants looked at the distractor before looking at the diamond (termed *distraction trials*), or if they did not respond quickly enough, the fruit reward was not delivered on that trial.

Bringing these ideas together, to effectively investigate whether reward-modulated attentional capture is mediated by a representation of outcome value we need a procedure in which (1) the critical reward-signalling stimuli are never targets of search; and (2) we change the value of the outcome signalled by a stimulus while keeping the identity of that outcome constant. The experiments presented here bridged this gap by incorporating instructed changes in outcome value—both increases and decreases—into a value-modulated attentional capture procedure based on the task used by Pearson et al. (2016). With regard to point (1) above, in this procedure there is always a cost in attending to the reward-signalling distractor: this item is never the target of search, and if participants look at it the reward is cancelled. So we can be confident that this task is measuring automatic patterns of reward-conditioned attentional capture, rather than top–down, strategic prioritisation. With regard to point (2), a key innovation of our *attentional revaluation task* (Figure 1) was to introduce “outcome” elements mediating between stimuli (colours) and rewards (points, corresponding to money). Specifically, the colour of a distractor signalled the type of fruit that could be won on the current trial, with different fruits having different point-values. The mediating fruit outcomes allowed us to keep the relationship between a stimulus and an outcome constant (e.g., a blue distractor might always signal that banana was available) while changing the value of that outcome by manipulating how many points each fruit was worth (for studies using a conceptually similar approach in the context of instrumental behaviour, see for example, Adams & Dickinson, 1981; de Wit et al., 2012; Luque et al., 2020; Tricomi et al., 2009).

Participants’ task was to earn as many points as possible (since points would later be converted to money) by making a rapid saccade to a diamond target among circles on each trial to win a fruit. In an initial training phase, one type of fruit was worth 500 points, and the other was worth 10 points. The colour of a distractor circle signalled which fruit was available: one colour (the *high-training distractor*) signalled that the high-value fruit was available; the other colour (*low-training distractor*) signalled the low-value fruit. If participants looked at the coloured distractor, the fruit available on that trial was cancelled; these were termed *distraction trials*. In line with prior findings, we expected more distraction trials when the display contained a high-training distractor than a low-training distractor.

Following this training phase, participants in Experiment 1a were told that the values of the fruits had changed: both fruits were now worth only 10 points, implementing a *devaluation* manipulation (cf. Adams & Dickinson, 1981). Conversely, participants in Experiment 1b were told that both fruits were now worth 500 points: a *super-valuation* manipulation. All participants then completed the test phase of the search task. The key question was how these changes in participants’ explicit knowledge of outcome value influenced patterns of attentional prioritisation that had previously formed during the training phase. Half of the participants in Experiments 1a and 1b completed the test phase under a “nominal extinction” procedure: participants could still earn fruits, but were not told the identity of the fruit earned on each trial. This nominal extinction approach is commonly used in studies of the effect of outcome devaluation on instrumental behaviour (e.g., de Wit et al., 2012; Gillan et al., 2015; Hogarth et al., 2007; Luque et al., 2017, 2020; Watson et al., 2018). Testing under extinction (i.e., without specific outcome feedback) ensures test-phase behaviour is based on previously acquired associations, independent of any impact of delivery of the revalued outcome itself. That is, omitting outcome feedback prevents new stimulus–reward learning from occurring during the test phase: it creates a situation in which behaviour could remain under the control of previously acquired knowledge but could no longer be influenced by direct experience of outcomes. The approach of making extinction “nominal”—that is, telling participants that they are still nevertheless earning outcomes/points during the test phase—is often used because it reduces the potential for a loss of responding that may otherwise occur if testing was conducted in “genuine” extinction, which could mask any impact of outcome revaluation.^1^ This approach let us examine whether the attentional prioritisation response *developed during training* was sensitive to changes in participants’ explicit knowledge of fruit values following instructed revaluation. If conditioned prioritisation was insensitive to the current value of the associated outcome, then we would expect the pattern learned in training (greater capture by the high-training than low-training distractor) to persist following devaluation (Experiment 1a) or super-valuation (Experiment 1b) during the test phase, despite the change in outcome values. By contrast, if prioritisation of the high-training distractor was mediated by a representation of the associated outcome—and hence dependent on the current value of that outcome—we would expect the pattern of bias to reflect changed values of the fruit outcomes, with both high-training and low-training distractors now equally likely to capture attention, consistent with the equal low (Experiment 1a) or high (Experiment 1b) value of these distractors during the test phase.

Data from participants tested under nominal extinction—termed the Dev group (Experiment 1a) and Super group (Experiment 1b)—let us assess whether conditioned prioritisation is mediated by explicit knowledge of outcome value, in the absence of further experience of stimulus–outcome (colour-fruit) pairings under the new value regime. The other half of participants in each experiment—DevFB and SuperFB groups, respectively—continued to receive trial-by-trial feedback on the identity of the fruit earned in each trial of the test phase. That is, in addition to knowledge of the revalued fruit outcomes, these participants had *direct experience* of the relationship between distractor colours and now-revalued fruits, allowing us to assess the impact of experience-driven training on updating of attentional priority following a change in outcome value. Thus data from groups given feedback during the test phase assessed whether previously established patterns of reward-conditioned attention would update in the face of further training under conditions in which these patterns of prioritisation no longer matched the prevailing stimulus–reward relationships.

## Experiment 1

### Method

#### Participants and apparatus

Previous studies have found medium to very large effects (*d_z_* = 0.54–2.20) for the influence of reward on attentional capture (Le Pelley et al., 2015; Pearson et al., 2016; Watson, Pearson, Most, et al., 2019). Hence we aimed to recruit at least 24 participants per condition; G*Power revealed that this would give power of .80 to detect a medium-sized effect (*d_z_* = 0.6) of reward on attention in each condition, and power >.90 to detect a medium-sized (η_p_^2^ = .06) interaction reflecting differences in this bias across conditions. In total, 51 UNSW Sydney students completed Experiment 1a (31 females; age *M* = 18.56, *SEM* = 0.19 years; Dev group *n* = 26, DevFB group *n* = 25), and 55 completed Experiment 1b (39 females; age *M* = 19.18, *SEM* = 0.39 years; Super group *n* = 28, SuperFB group *n* = 27). Group assignment alternated based on order of arrival. Participants earned course credit, and received a monetary bonus depending on points earned in the attentional revaluation task (*M* = AU$9.91, *SEM* = AU$0.16). All research reported in this article was approved by the UNSW Human Research Ethics Advisory Panel (Psychology); experiment scripts and data are available at https://osf.io/nuaxg.

Stimuli were presented on a 23-in monitor (60 Hz refresh, 1,920 × 1,080 resolution), controlled by MATLAB with Psychophysics Toolbox extensions (Kleiner et al., 2007). Participants were tested using a Tobii Pro Spectrum eye-tracker (sampling rate 600 Hz). Gaze data were down-sampled to 100 Hz for gaze-contingent calculations during stimulus presentation. Head position was stabilised using a chin-rest 60 cm from the monitor.

#### Design and procedure

The attentional revaluation task consisted of four components: the initial value instructions, training phase, revaluation instructions, and test phase.

##### Initial value instructions

Participants were told their aim was to earn points (which would later be converted into money), and that they could win points by earning fruits—lemons and bananas. For half of the participants, lemons were initially described as being worth 500 points and bananas 10 points; for remaining participants this was reversed. Participants were told they could earn fruits by looking at the diamond target “as quickly and directly as possible,” but that if they looked at the coloured circle in the search display, the fruit they could have earned would be cancelled. Participants were not informed of the specific colour–fruit contingencies (e.g., that a blue circle signalled availability of lemons and an orange circle signalled bananas).

##### Training phase

Each trial of the search task consisted of a fixation display, search display, and feedback display (Figure 1). All stimuli appeared on a black background. The fixation display comprised a central white cross surrounded by a white circle (diameter 3.0° visual angle). Once 700 ms of gaze time had accumulated inside this circle, or after 4,000 ms, the cross and circle turned yellow. After 300 ms the screen blanked, and 150 ms later the search display appeared: a diamond and 5 circles, each 2.3 × 2.3°, distributed evenly around screen centre at an eccentricity of 5.1°. One of the circles—the *distractor*—was either orange (CIE *x*/*y* chromaticity coordinates .493/.445) or blue (CIE *x*/*y* .192/.216) with similar luminance (~24.5 cd/m^2^). All other shapes were grey (CIE *x*/*y* .327/.400, luminance ~8.3 cd/m^2^).

A response was registered when participants had accumulated 100 ms of gaze dwell time within a region of diameter 3.5° centred on the diamond target. The colour of the distractor signalled the type of fruit available for a rapid response: for half of the participants, a blue distractor signalled availability of a lemon, and an orange distractor signalled a banana; for the remaining participants this assignment was reversed. The distractor signalling the high-value fruit (worth 500 points) was termed the *high-training distractor*, and the distractor signalling the low-value fruit (worth 10 points) was the *low-training distractor*. If any gaze fell within a region of diameter 5.1° centred on the distractor prior to a response being registered, it was recorded as a *distraction trial* and no reward was given.

The feedback display appeared when a response was registered, or after 2,000 ms (timeout). If response time was below 1,000 ms and it was not a distraction trial, feedback stated “Fruit won!” with a picture of the appropriate fruit. If the trial was a distraction trial, feedback stated “No reward: You could have won:,” and showed the fruit overlaid with a red “X.” If response time was above 1,000 ms, feedback stated “Too slow: You could have won:,” and presented the fruit overlaid with a red “X.” If no response was made before the trial timed-out, feedback read: “Too slow: Please try to look at the diamond more quickly.” Feedback appeared for 1,400 ms; the next trial then began after a 1,400 ms blank interval.

There were 16 blocks of trials in the training phase, each containing 24 trials: 12 with a high-training distractor, and 12 with a low-training distractor, in random order. Target and distractor location were randomly determined on each trial. Participants took a break after each block, during which they saw a reminder of the fruit values (as in Figure 1a) on-screen for at least 10 s; participants then opted when to continue with the task.

##### Revaluation and feedback instructions

Immediately following the training phase, participants received instructions regarding the test phase (see Table 1). In Experiment 1a, participants were told that the fruit previously worth 500 points in training was now worth 10 points, while the fruit previously worth 10 points was still worth 10 points. In Experiment 1b, the fruit previously worth 10 points in training was now worth 500 points, while the fruit previously worth 500 points was still worth 500 points.

**Table 1.** Design of each experiment for one counterbalance condition (where lemons were the high-value fruit in training, and the high-training distractor was blue).

Experiment	Group	Initial value instructions	Training phase	Revaluation instructions	Test phase
Exp 1a	Dev	Lemon = 500 pts Banana = 10 pts	Blue→Lemon Orange→Banana	Lemon = 10 pts Banana = 10 pts	Blue→?? Orange→??
	DevFB				Blue→Lemon Orange→Banana
Exp 1b	Super	Lemon = 500 pts Banana = 10 pts	Blue→Lemon Orange→Banana	Lemon = 500 pts Banana = 500 pts	Blue→?? Orange→??
	SuperFB				Blue→Lemon Orange→Banana
Exp 2	NoRev	Lemon = 500 pts Banana = 10 pts	Blue→Lemon Orange→Banana	Lemon = 500 pts Banana = 10 pts	Blue→?? Orange→??
	Rev			Lemon = 10 pts Banana = 500 pts	Blue→?? Orange→??
	RevFB			Lemon = 10 pts Banana = 500 pts	Blue→Lemon Orange→Banana

Colours refer to colours of the distractor in the search display; fruits refer to outcomes that could be won. Note: fruit–value and colour–fruit contingencies were counterbalanced across participants.

After this instruction, all participants answered check questions to ensure their knowledge of the current fruit values: participants were shown the picture of a lemon and a banana and were asked to select the current value of each fruit. Both responses had to be correct before they could proceed.

Participants in the Dev (Experiment 1a) and Super (Experiment 1b) group were informed that while they would still be earning fruits during the test phase, they would no longer be told if they had earned a lemon or banana on each trial; “instead you will simply be told whether or not you won a fruit—and we will keep track of how many points you have earned.” Participants in the DevFB and SuperFB groups were told that “as before, you will be told whether you earned a lemon or a banana on each trial, and we will keep track of how many points you have earned.”

##### Test phase

During the subsequent test phase of the search task, participants in the Dev and Super groups continued to earn fruits for rapid responses, but were not told the identity of the fruit earned on each trial. Feedback for these participants was as for the training phase, but “??” appeared where a picture of the fruit had appeared during training. The DevFB and SuperFB groups continued to receive feedback on the identity of the fruit earned—or omitted—on each trial (lemon or banana), as in training. Participants completed 8 blocks of trials in the test phase, with blocks structured as in training. All participants were reminded of the current fruit values in the break that followed each block.

##### Knowledge checks

Following the test phase, participants’ knowledge of the colour–fruit contingencies was assessed. Participants were told that the type of fruit that could be won on each trial depended on the colour of the coloured circle in the search display. They were then presented with an orange and a blue circle, in random order, and were asked to select which fruit (banana or lemon) they could win when that stimulus appeared in the search display. A final knowledge check of fruit values verified that participants had retained knowledge of the updated fruit values following revaluation: each fruit appeared in random order and participants selected whether it was currently worth 500 points or 10 points.

#### Data preparation

Screening of data from the search task followed prior protocols (e.g., Le Pelley et al., 2015; Pearson et al., 2016). We discarded data from the first two trials after each break, trials timing out with no response (0.58% of all trials in Experiment 1a; 1.07% in Experiment 1b), and trials with <25% valid gaze data (as a result of blinks etc.: 0.55% of trials in Experiment 1a; 0.30% in Experiment 1b). Our primary dependent variable was the *proportion of distraction trials*: the proportion of trials on which participants looked at the coloured distractor, cancelling the outcome. We analysed proportion of distraction trials as a function of whether the trial featured a high- or low-training distractor; note that we label distractors according to the value they signalled *during the training phase*.

In line with previous work (e.g., Pearson et al., 2016; Watson et al., 2020), we also analysed the direction of the first saccade on each trial as a function of the latency of that saccade (i.e., time between display onset and initiation of the first saccadic eye movement). A velocity-threshold identification algorithm (Salvucci & Goldberg, 2000) identified saccades using raw gaze data. Gaps in the data shorter than 75 ms were first interpolated using linear interpolation. Gaze data were then smoothed using a five-point moving average filter. The first saccade on each trial was then identified as the first eye movement remaining above a velocity of 40° visual angle per second for at least 10 ms. This saccade was classified as moving in the direction of the distractor if the saccade vector had an angular deviation less than 30° to the left or right of the centre of the distractor.

For latency-based analyses, trials were excluded if the saccade start point was not within 100 pixels of the central fixation point, if saccade latency was below 80 ms, if gaps in the gaze data were too large to be interpolated, or if there was insufficient gaze data to identify a saccade. Any participant with >30% of invalid trials in a given phase of the task (training or test) was excluded from latency-based analyses of that phase (see Supplementary Table S1 for numbers of retained participants and trials). Included trial data for each participant were grouped by phase (training vs. test) and distractor-type (high- vs. low-training). The Vincentising procedure (Ratcliff, 1979) was then used to separate first saccade latencies into three time bins (defined by the tertiles of the distribution) representing the fastest, middle, and slowest groups of saccades. For each time bin in each phase, we calculated the proportion of first saccades that went towards the distractor.

### Results

#### Experiment 1a

##### Proportion of distraction trials

We first examined the proportion of distraction trials across the task via a 2 (phase: training vs. test) × 2 (distractor-type: high- vs. low-training) × 2 (group: Dev vs. DevFB) ANOVA; phase and distractor-type were repeated measures, and group was a between-subjects factor. This revealed a main effect of distractor-type, *F*(1,49) = 32.8, *p* < .001, η_p_^2^ = .40 [.22, .53],^2^ with participants more likely to look at the high-training distractor than the low-training distractor; an effect that did not interact significantly with phase, *F*(1,49) = 1.88, *p* = .18, η_p_^2^ = .04 [0, .15]. The three-way interaction was also nonsignificant, *F*(1,49) < 0.001, *p* = .98, η_p_^2^ < .001. Nevertheless, planned analyses focused on the training and test phases separately.

##### Training phase

**Figure 2.**
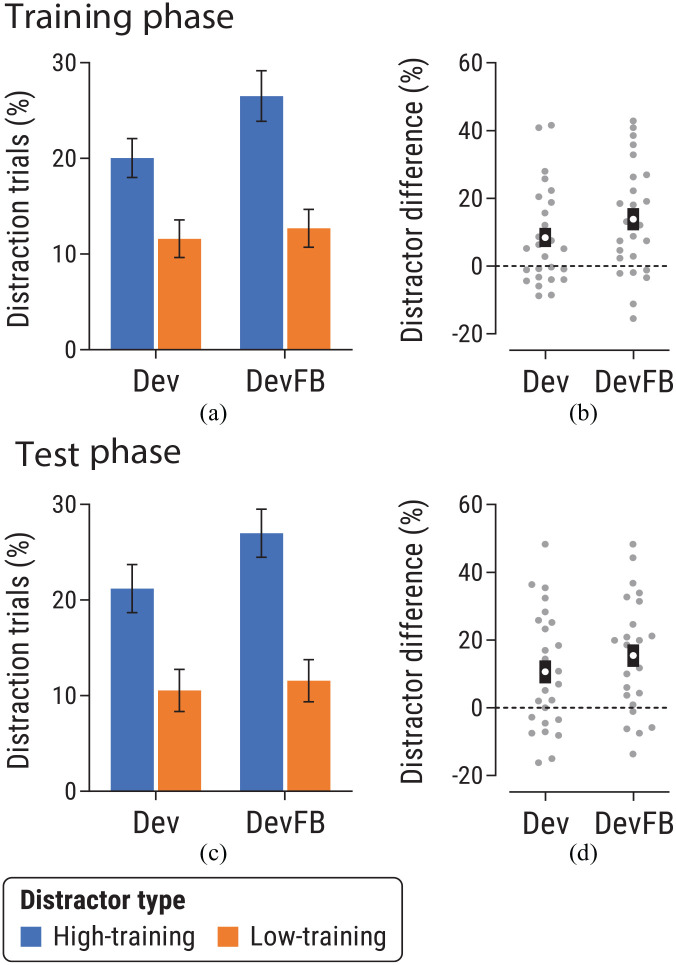
Data from the (a, b) training and (c, d) test phases of Experiment 1a, for Dev and DevFB groups. Panels (a) and (c) show the proportion of distraction trials for trials featuring a high-training or low-training distractor. Note that distractors are defined by the value of the outcome they signalled during the training phase. Error bars show within-subjects *SEM* (Morey, 2008). Panels (b) and (d) show the difference in proportion of distraction trials for high-training versus low-training distractors for each group. Grey points show individual data; the white dot indicates the mean and the surrounding black region shows *SEM*.

We analysed proportion of distraction trials during the training phase (Figure 2a) via ANOVA with factors of distractor-type and group. There was a significant main effect of distractor-type, *F*(1,49) = 26.72, *p* < .001, η_p_^2^ = .35 [.18, .49]: participants were more likely to look at the distractor signalling availability of a high-value fruit versus a low-value fruit, even though this was counterproductive because looking at the distractor caused cancellation of the fruit. There was no main effect of group, *F*(1,49) = 1.64, *p* = .21, η_p_^2^ = .03 [0, .14], or interaction, *F*(1,49) = 1.57, *p* = .22, η_p_^2^ = .03 [0, .14]. These latter null findings are unsurprising, since both groups received equivalent treatment until after the training phase.

The analyses described above collapsed across training blocks, including initial blocks where reward-related effects were small: analysis as a function of block (see Supplementary Materials) showed that the effect of distractor-type increased over the course of training, as expected for a learned effect. Analysis of data from the final two training blocks (i.e., immediately prior to the value-switch manipulation) showed a similar pattern to the whole-phase analysis: a main effect of distractor-type that did not interact with group (see Supplementary Materials).

##### Test phase

Figure 2c shows data from the test phase, following devaluation of the high-value fruit. ANOVA found a main effect of distractor-type, *F*(1,49) = 30.99, *p* < .001, η_p_^2^ = .39 [.21, .52], with participants more likely to look at high-training than low-training distractors, even though both outcomes had the same (low) value during the test phase. There was no main effect of group, *F*(1,49) = 2.04, *p* = .16, η_p_^2^ = .04 [0, .16]. Importantly, the distractor-type × group interaction was not significant, *F*(1,49) = 1.24, *p* = .27, η_p_^2^ = .025 [0, .13].

To further analyse this nonsignificant interaction, we calculated distractor difference scores for each participant by taking the difference in proportion of distraction trials between high- and low-training distractor trials (Figure 2d). Comparing these scores between Dev and DevFB groups via a Bayesian *t*-test (using the default prior in JASP: JASP Team, 2020) yielded a Bayes factor of *BF*_01_ = 2.15 in favour of the null hypothesis. Notably, Figure 2b shows that the mean attentional bias was numerically smaller in the Dev group than the DevFB group during the training phase, a pattern that persisted in the test phase. This implies that any small between-group difference in bias during the test phase was not a consequence of the difference in their treatment. In line with this idea, comparing test-phase distractor difference scores while controlling for each group’s attentional bias during training (by using scores from the training phase as a covariate in a Bayesian one-way ANCOVA), yielded *BF*_01_ = 3.48, suggesting moderate evidence in favour of the null hypothesis of no difference between groups during the test phase (Lee & Wagenmakers, 2013).

Analyses of simple effects tested the effect of distractor type in each group during the test phase. In both groups participants were significantly more likely to look at the high-training distractor than the low-training distractor: Dev group, *t*(25) = 3.14, *p* = .004, *d_z_* = .62 [0.19, 1.03]; DevFB group, *t*(24) = 4.75, *p* < .001, *d_z_* = .95 [0.47, 1.41].

Analysis of data from the test phase as a function of block (see Supplementary Materials) supported the above findings, with little evidence for a change in the pattern of reward-related attentional bias across the course of the test phase.

##### Latency-based analyses

**Figure 3.**
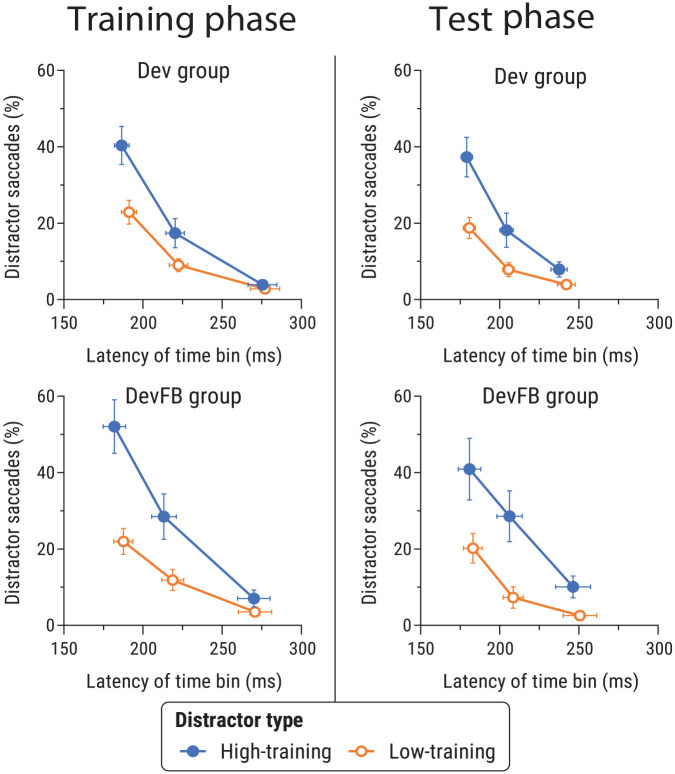
Proportion of saccades going towards the high- and low-training distractors as a function of mean saccade latency in the training phase (left panels) and the test phase (right panels) of Experiment 1a, for the Dev group (top panels) and the DevFB group (bottom panels). Mean proportion of saccades and saccade latency were calculated separately for each of three time bins of individual-participant saccade latency distributions—fastest, middle, and slowest, defined by the tertiles of the distribution—and the mean data for each time bin are shown as points in the plots. Error bars show *SEM*.

Figure 3 shows the proportion of first saccades that went towards distractors as a function of saccade latency. We analysed data for each phase using separate 2 (distractor-type: high- vs. low-training distractor) × 2 (group: Dev, DevFB) × 3 (latency time bin: fastest, middle, slowest) ANOVAs.

##### Training phase

In the training phase, there was a significant main effect of distractor-type, *F*(1,42) = 27.70, *p* < .001, η_p_^2^ = .40 [.20, .54], with more first saccades towards the high-training distractor than the low-training distractor. There was also a main effect of saccade latency time bin, *F*(2,84) = 111.6, *p* < .001, η_p_^2^ = .73 [.64, .78], with shorter-latency saccades being more likely to go towards distractors. The interaction between these two factors was also significant, *F*(2,84) = 17.0, *p* < .001, η_p_^2^ = .29 [.15, .40], with the bias towards the high-training distractor over the low-training distractor being more pronounced at shorter saccade latencies. There were no significant effects involving group, *F*s < 2.51, *p*s > .12, η_p_^2^s < .056.

##### Test phase

In the test phase, ANOVA again revealed significant main effects of distractor-type, *F*(1,36) = 19.7, *p* < .001, η_p_^2^ = .35 [.15, .51], and latency bin, *F*(2,72) = 72.0, *p* < .001, η_p_^2^ = .66 [.55, .73], and a significant distractor-type × latency bin interaction, *F*(2,72) = 6.43, *p* = .003, η_p_^2^ = .15 [.03, .26], with a bias towards the high-training distractor over the low-training distractor that was more pronounced at shorter saccade latencies. There were no significant effects involving group, *F*s < 0.81, *p*s > .37, η_p_^2^s < .022.

Prior studies have shown that the influence of reward on gaze is most apparent among participants’ fastest saccades (Failing et al., 2015; Pearson et al., 2016). Consequently, follow-up analyses focused on data from the fastest saccade latency time bin. Paired sample *t-*tests revealed a significant bias towards the high-training distractor (vs. the low-training distractor) among these rapid saccades in both the Dev group, *t*(21) = 2.76, *p* = .012, *d_z_* = .59 [0.13, 1.04], and the DevFB group, *t*(15) = 2.04, *p* = .030, *d_z_* = .60 [0.06, 1.12].

A 2 × 2 ANOVA comparing the Dev and DevFB groups in the proportion of saccades made to the high- and low-training distractors in the fastest latency bin revealed a significant main effect of distractor-type, *F*(1,36) = 13.22, *p* < .001, η_p_^2^ = .27 [.08, .43]. The main effect of group was not significant, *F*(1,36) = 0.26, *p* = .61, η_p_^2^ = .007 [0, .11], nor was the group × distractor-type interaction, *F*(1,36) = 0.04, *p* = .84, η_p_^2^ < .001 [0, .04]. Thus the pattern of performance for the fastest saccades did not differ significantly between the two groups.

##### Knowledge checks

Participants completed knowledge checks following the search task to ensure they had understood and retained revaluation instructions. All participants correctly identified the current value of each fruit.^3^ We also assessed participants’ knowledge of the colour–fruit contingencies (i.e., which fruit was signalled by each distractor colour). In each group, only two participants failed to correctly identify the colour–fruit contingencies. Patterns of significant findings were unchanged by exclusion of these participants.

#### Experiment 1b

##### Proportion of distraction trials

Analysis of the proportion of distraction trials across Experiment 1b using a 2 (phase) × 2 (distractor-type) × 2 (group: Super vs. SuperFB) ANOVA revealed a main effect of distractor-type, *F*(1,53) = 13.9, *p* < .001, η_p_^2^ = .21 [.07, .35], with participants more likely to look at the high-training distractor than the low-training distractor; an effect that did not interact significantly with phase, *F*(1,53) = 0.28, *p* = .598, η_p_^2^ = .005 [0, .08]. The three-way interaction was also nonsignificant, *F*(1,53) = 0.006, *p* = .94, η_p_^2^ < .001 [0, .005]. Planned analyses focused on data from training and test phases separately.

##### Training phase

**Figure 4.**
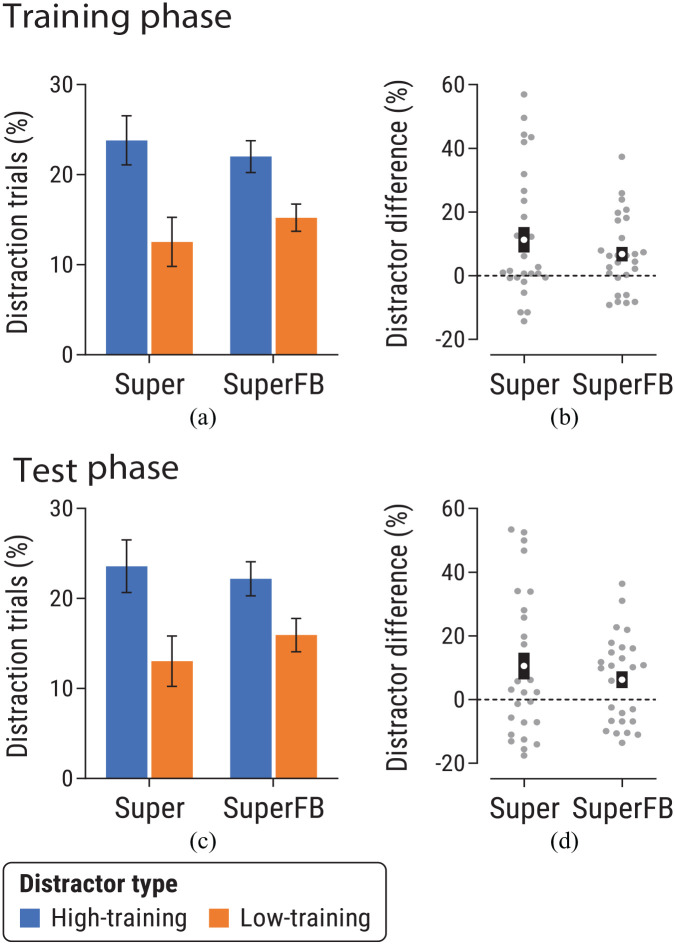
Data from the (a, b) training and (c, d) test phases of Experiment 1b, for Super and SuperFB groups. Panels (a) and (c) show the proportion of distraction trials for trials featuring a high-training or low-training distractor. Panels (b) and (d) show the difference in proportion of distraction trials for high-training versus low-training distractors for each group. See caption of Figure 2 for further details.

A distractor-type × group ANOVA revealed a main effect of distractor-type in the training phase, *F*(1,53) = 15.18, *p* < .001, η_p_^2^ = .22 [.08, .37], with more distraction trials for the high-training than low-training distractor (Figure 4a). There was no main effect of group, *F*(1,53) = 0.04, *p* = .84, η_p_^2^ < .001 [0, .03], or interaction, *F*(1,53) = 0.93, *p* = .34, η_p_^2^ = .02 [0, .11]. Repeating these analyses with data from only the last two training blocks did not affect the pattern of significant findings (see Supplementary Materials).

##### Test phase

Figure 4c shows data from the test phase, following super-valuation of the low-value fruit (see Supplementary Materials for analysis of data as a function of blocks in the test phase). ANOVA found a main effect of distractor-type, *F*(1,53) = 11.33, *p* = .001, η_p_^2^ = .18 [.05, .32], with participants more likely to look at high-training than low-training distractors, even though both outcomes had the same (high) value during the test phase. There was no main effect of group, *F*(1,53) = 0.12, *p* = .73, η_p_^2^ = .002 [0, .06]. Importantly, the group × distractor-type interaction was not significant, *F*(1,53) = 0.74, *p* = .39, η_p_^2^ = .01 [0, .10].

Following up this nonsignificant interaction, Bayesian analysis of distractor difference scores from the test phase (Figure 4d) yielded *BF*_01_ = 2.71 in favour of the null hypothesis. Further controlling for attentional bias during the training phase (by using distractor difference scores from the training phase as a covariate in a Bayesian one-way ANCOVA) yielded *BF*_01_ = 4.57, suggesting moderate evidence in favour of the null hypothesis of no difference between groups during the test phase.

Analyses of simple effects tested the effect of distractor type in each group during the test phase. In both groups, participants were significantly more likely to look at the high-training distractor than the low-training distractor: Super group, *t*(27) = 2.52, *p* = .018, *d_z_* = .48 [0.08, 0.86]; SuperFB group, *t*(26) = 2.37, *p* = .025, *d_z_* = .46 [0.06, 0.85].

##### Latency-based analyses

**Figure 5.**
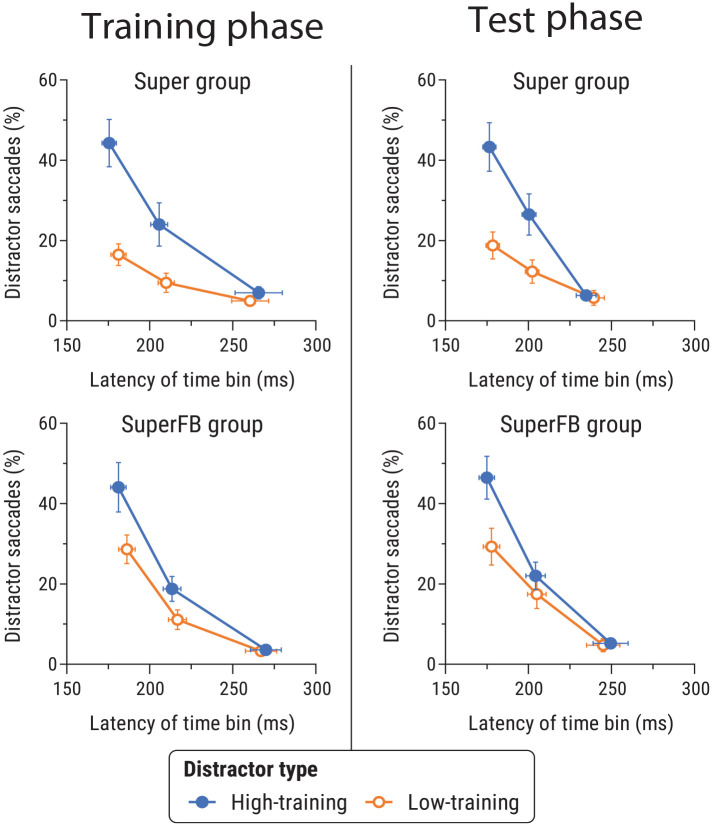
Proportion of saccades going towards the high- and low-training distractors as a function of mean saccade latency in the training phase (left) and the test phase (right) of Experiment 1b, for the Super group (top) and the SuperFB group (bottom). See caption of Figure 3 for further details.

Figure 5 shows the proportion of first saccades that went towards distractors as a function of saccade latency in Experiment 1b. We analysed data for each phase separately.

##### Training phase

In the training phase, there were significant main effects of distractor type, *F*(1,38) = 15.26, *p* < .001, η_p_^2^ = .29 [.10, .45], and latency bin, *F*(2,76) = 111.4, *p* < .001, η_p_^2^ = .75 [.66, .79], and a significant distractor-type × latency bin interaction, *F*(2,76) = 17.65, *p* < .001, η_p_^2^ = .32 [.17, .43]. No other effects involving group were significant, *F*s < 2.96, *p*s > .058, η_p_^2^s < .07.

##### Test phase

In the test phase, ANOVA again revealed main effects of distractor-type, *F*(1,40) = 9.39, *p* = .004, η_p_^2^ = .19 [.04, .35], and latency bin, *F*(2,80) = 107.4, *p* < .001, η_p_^2^ = .73 [.64, .78], and a significant distractor-type × latency bin interaction, *F*(2,80) = 13.0, *p* < .001, η_p_^2^ = .25 [.11, .36]. There were no significant effects involving group, *F*s < 2.25, *p*s > .11, η_p_^2^s < .053.

Follow-up analyses restricted to the fastest saccade latency time bin revealed a significant effect of distractor type in both the Super group, *t*(23) = 3.20, *p* = .004, *d_z_* = .65 [0.21, 1.09], and the SuperFB group, *t*(17) = 2.30, *p* = .034, *d_z_* = .54 [0.04, 1.03]. A 2 × 2 ANOVA comparing the Super and SuperFB groups revealed a significant main effect of distractor-type, *F*(1,40) = 14.48, *p* < .001, η_p_^2^ = .27 [.09, .42], but no main effect of group, *F*(1,40) = 2.24, *p* = .14, η_p_^2^ = .05 [0, .19], and no interaction, *F*(1,40) = 0.45, *p* = .50, η_p_^2^ = .01 [0, .11]. Thus the pattern of performance for the fastest saccades did not differ significantly between the two groups.

##### Knowledge checks

All participants correctly identified the current value of each fruit in the knowledge check. Five participants in the Super group and three in the SuperFB group failed to correctly identify the colour–fruit contingencies. Patterns of significant findings were unchanged by exclusion of these participants.

#### Combined analysis of Experiments 1a and 1b

To increase power, a final set of analyses combined the data from Experiments 1a and b to examine the influence of outcome revaluation—regardless of whether this was devaluation (Experiment 1a) or super-valuation (Experiment 1b)—on reward-related attentional bias. An initial ANOVA including Experiment as a factor found that it did not exert a main effect or interact with any other factor (smallest *p* = .129) so for simplicity we collapsed across experiments in subsequent analyses. Findings mirrored those of the individual experiments, though with somewhat more decisive results as a consequence of the larger, pooled sample (*N* = 106). ANOVA with factors of phase, distractor-type, and group (tested under extinction vs. tested with feedback) revealed a main effect of distractor type, *F*(1,104) = 42.3, *p* < .001, η_p_^2^ = .29 [.17, .39], with a general bias towards the high-training distractor over the low-training distractor that did not interact with phase, *F*(1,104) = 0.53, *p* = .468, η_p_^2^ = .005 [0, .05]. ANOVA restricted to the test phase revealed a main effect of distractor-type, *F*(1,104) = 37.9, *p* < .001, η_p_^2^ = .27 [.15, .37], that did not interact with group, *F*(1,104) = 0.008, *p* = .926, η_p_^2^ < .001 [0, .003]. A Bayesian analysis of distractor difference scores from the test phase revealed moderate evidence for the null effect of group, BF_01_ = 4.85. The strength of support for the null rose further when using Bayesian ANCOVA to control for attentional bias during the training phase, BF_01_ = 8.22.

### Discussion

We investigated whether conditioned attentional prioritisation of reward-related stimuli was sensitive to acute changes in the values of outcomes. In the training phase, participants were more often distracted by a stimulus signalling a high-value outcome versus a low-value outcome—even though looking at the high-training distractor was counterproductive to participants’ goal of maximising their payoff, as it resulted in cancellation of a larger reward (relative to looking at the low-training distractor). Latency-based analyses of saccade data showed that this pattern of greater attentional capture by high-reward versus low-reward distractors was particularly pronounced among participants’ fastest saccades. These findings are consistent with previous demonstrations of value-modulated attentional capture (e.g., Koenig et al., 2017; Le Pelley et al., 2015; Pearson et al., 2016).

Following this training phase, instructed devaluation rendered both fruits of equally low (Experiment 1a) or high (1b) value. Participants in both experiments nevertheless continued to show an attentional bias towards the high-training distractor relative to the low-training distractor. Providing trial-by-trial feedback on the identity of the fruit earned during the test phase (groups DevFB and SuperFB) also did not result in a change in attentional prioritisation of distractors in line with their current value.

The failure to update patterns of attentional priority in Experiment 1 cannot simply be ascribed to devaluation being ineffective in changing outcome values. We verified that participants were explicitly aware of the new values of the fruits in the test phase: they were informed of the change, passed check questions confirming their understanding of the new values, were reminded at the end of each block, and all participants correctly reported these values following the task. Participants clearly had explicit knowledge of the revised fruit values, and knew which distractor earned which fruit, and yet their pattern of attention did not change—even in the face of additional, direct experience of pairings of distractors with revalued outcomes (cf. Adams, 1982; Adams & Dickinson, 1981). This dissociation of knowledge and performance implies that there are conditions under which participants will not update a pattern of reward-related attentional priority even though it is a poor match to prevailing stimulus–reward contingencies: we return to this idea in the “General discussion” section.

The findings from the Dev group of Experiment 1a mirror those of a recent study by Watson et al. (2022). Using a similar approach, but with food rather than monetary rewards, Watson et al. showed that devaluation of a food outcome (through feeding participants on that food to satiety) did not reduce attentional capture by a signal of the now-devalued food when tested under nominal extinction. Experiment 1 extends these prior results in important ways. First, data from the DevFB group show that attentional bias following devaluation persists despite direct experience of stimulus–outcome pairings under the new value regime, underlining the resistance to updating attentional priorities. Second, our data rule out the possibility that earlier findings of persistence were a consequence of the specific scenario wherein—following devaluation—no substantial rewards were available (as all outcomes now had low value), such that capture by distractors became unimportant. Experiment 1b found evidence of similar persistence even after super-valuation meant that all outcomes had high value, so capture remained meaningful for participants’ earnings.

## Experiment 2

Experiment 1 found no evidence of a change in participants’ knowledge of outcome values on a previously established pattern of reward-conditioned attentional bias. One notable aspect of the revaluation procedure used in Experiment 1 is that it rendered both outcomes of equal value in the test phase: either low (Experiment 1a) or high (Experiment 1b). This equivalence of outcome values may have meant that participants did not strive to earn one particular fruit over the other, which may in turn have limited their motivation to engage attentional control processes so as to change pre-existing patterns of attentional bias. Experiment 2 addressed this issue by using a procedure in which the values of the two outcomes were *reversed* following training: the (previously) high-value fruit was thereafter worth 10 points, and the low-value fruit was worth 500 points (see Table 1). Hence there was a difference in the relative value of outcomes during the test phase, which may have provided greater incentive for participants to exert the cognitive resources needed to update attentional control settings to reflect the changed outcome values. Participants in the Rev group were tested under nominal extinction, whereas the RevFB group continued to receive outcome feedback in the test phase. Experiment 2 also included a group that did not undergo revaluation of the outcomes following the training phase (NoRev), providing a baseline against which to assess the effect of revaluation in the other groups (as opposed to comparing performance before and after revaluation as in Experiment 1).

### Method

#### Participants and apparatus

Recruitment was as in Experiment 1; a total of 86 participants completed Experiment 2 (64 females; age *M* = 19.53, *SEM* = 0.32 years; NoRev group *n* = 28; Rev group *n* = 29; RevFB group *n* = 29). The first 34 participants were tested using Tobii TX300 eye-trackers (sampling rate 300 Hz); for subsequent participants we used Tobii Pro Spectrum eye-trackers.^4^ Preliminary analyses indicated that the model of eye tracker had no significant effect on findings, so we collapsed across this factor in analyses reported here.

#### Design, procedure, and data preparation

All aspects were as for Experiment 1, with exceptions noted here. In the instructions following the training phase, participants in Rev and RevFB groups were told that the fruit previously worth 500 points in training was now worth 10 points, and the fruit previously worth 10 points was now worth 500 points. Participants in the NoRev group were simply reminded of the values of the fruits, which were unchanged from training. Participants in the RevFB group continued to receive feedback on the identity of the fruit earned on each trial of the test phase; participants in the Rev and NoRev group completed the test phase under nominal extinction. Knowledge checks at the end of the experiment were as in Experiment 1, with the exception that the final test of value knowledge (verifying participants’ knowledge of the current value of each of the fruit outcomes) was omitted: data from Experiment 1 showed that every participant could correctly identify fruit values, so it seems safe to assume that they would also have been able to do so in Experiment 2. As in Experiment 1, we discarded data from the two trials after each break, trials timing out with no response (1.46% of all trials), and trials with <25% valid gaze data (0.42%). Latency-based analyses had additional screening (see Experiment 1): Table S1 (Supplementary Materials) shows numbers of retained participants and trials.

### Results

#### Proportion of distraction trials

Analysis of data across Experiment 2 using 2 (phase: training vs. test phase) × 2 (distractor-type: high- vs. low-training distractor) × 3 (revaluation group: NoRev, Rev, RevFB) ANOVA revealed a significant three-way interaction, *F*(2,83) = 11.57, *p* < .001, η_p_^2^ = .22 [.09, .33], suggesting a difference in the distractor-type × group interaction between the training and test phases. Planned follow-up analyses focused on training and test phases separately.

##### Training phase

**Figure 6.**
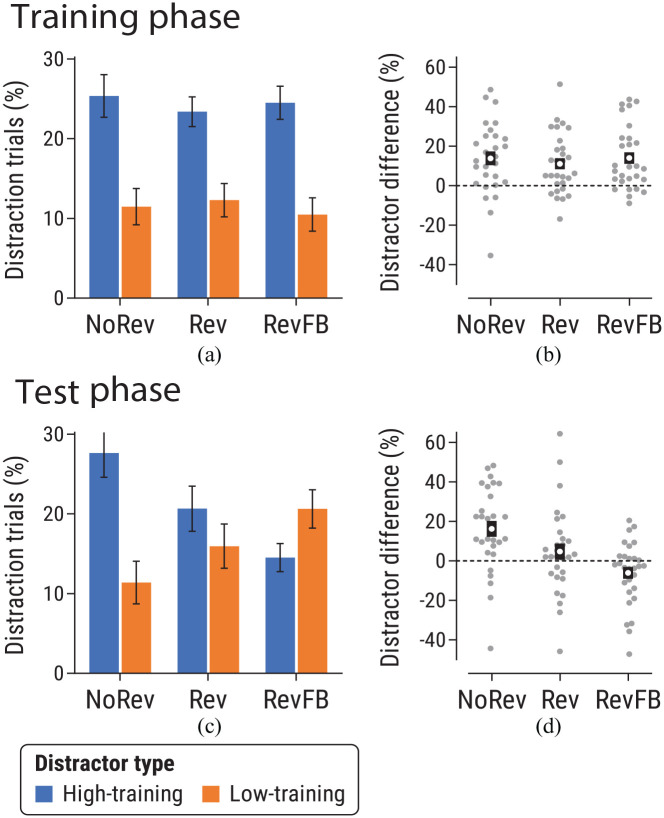
Data from the (a, b) training and (c, d) test phases of Experiment 2, for NoRev, Rev and RevFB groups. Panels (a) and (c) show the proportion of distraction trials for trials featuring a high-training or low-training distractor. Panels (b) and (d) show the difference in proportion of distraction trials for high-training versus low-training distractors for each group. See caption of Figure 2 for further details.

A 2 (distractor-type) × 3 (revaluation group) ANOVA found a main effect of distractor-type during training, *F*(1,83) = 52.75, *p* < .001, η_p_^2^ = .39 [.25, .50], with more distraction by high-training than low-training distractors (see Figure 6a). There was no effect of revaluation group, *F*(2,83) = 0.07, *p* = .93, η_p_^2^ = .002 [0, .02], or revaluation group × distractor-type interaction, *F*(2,83) = 0.28, *p* = .75, η_p_^2^ = .007 [0, .04]. Analysis restricted to the final two training blocks found the same pattern of significant findings (see Supplementary Materials).

##### Test phase

Analysis of the test phase (Figure 6c) found a main effect of distractor-type, *F*(1,83) = 5.21, *p* = .025, η_p_^2^ = .06 [.003, .15], while the main effect of revaluation group was not significant, *F*(2,83) = 0.27, *p* = .76, η_p_^2^ = .006 [0, .04]. Critically, a significant revaluation group × distractor-type interaction, *F*(2,83) = 8.81, *p* < .001, η_p_^2^ = .18 [.06, .28], indicated different patterns of attentional bias across the three groups.

To decompose this interaction, we first examined simple effects in each group. In the NoRev group, there was a significant effect of distractor-type, *t*(27) = 3.99, *p* < .001, *d_z_* = 0.75 [0.33, 1.17], with participants more likely to look at the high-training than the low-training distractor (see Figure 6d). Thus for group NoRev, the bias established during training persisted in the test phase, even under nominal extinction. By contrast, in the Rev group, there was no significant effect of distractor type, *t*(28) = 1.14, *p* = .26, *d_z_* = 0.21 [−0.16, 0.58]. A Bayesian *t-*test was used to assess evidence for the one-tailed alternative hypothesis of greater capture by the low-training than the high-training distractor in this group (the pattern expected if attention were mediated by current outcome value). The resulting *BF*_01_ = 9.97 represents moderate-to-strong evidence in favour of the null hypothesis. Finally, participants in the RevFB group were more likely to look at the low-training distractor than the high-training distractor; this difference came close to the threshold of significance, *t*(28) = 2.04, *p* = .051, *d_z_* = 0.38 [−0.001, 0.75].

We next compared between-group differences in attention by examining the interaction between revaluation group and distractor-type. The Rev and NoRev groups differed only in whether the values of the fruits changed from training to test (neither group received outcome feedback during test). Hence contrasting these two groups indexed the effect of outcome revaluation on attention. A 2 × 2 ANOVA revealed that the revaluation group × distractor-type interaction approached significance, *F*(1,55) = 3.97, *p* = .051, η_p_^2^ = .07 [0, .19], with a trend towards a greater difference in capture by high-training versus low-training distractors in the NoRev group than the Rev group (Figure 6d).

Our second comparison of interest was between Rev and RevFB groups, which differed only in whether outcome feedback was provided during the test phase (both groups experienced revaluation). Contrasting these two groups thus indexed the effect of feedback following outcome revaluation. ANOVA found a significant revaluation group × distractor-type interaction, *F*(1,56) = 4.52, *p* = .038, η_p_^2^ = .08 [.002, .20], with a greater difference in capture by high- versus low-training distractors in the RevFB group than the Rev group (Figure 6d).

Finally, ANOVA comparing NoRev and RevFB groups revealed a significant interaction, *F*(1,56) = 19.76, *p* < .001, η_p_^2^ = .26 [.11, .40], in line with the very different pattern of attentional bias observed during the test phase in these groups.

Analysis of data from the test phase as a function of block (see Supplementary Materials) suggested that patterns of attentional bias adapted rapidly in the groups undergoing revaluation, with little evidence of further systematic change following the first block of the test phase.

#### Latency-based analyses

**Figure 7.**
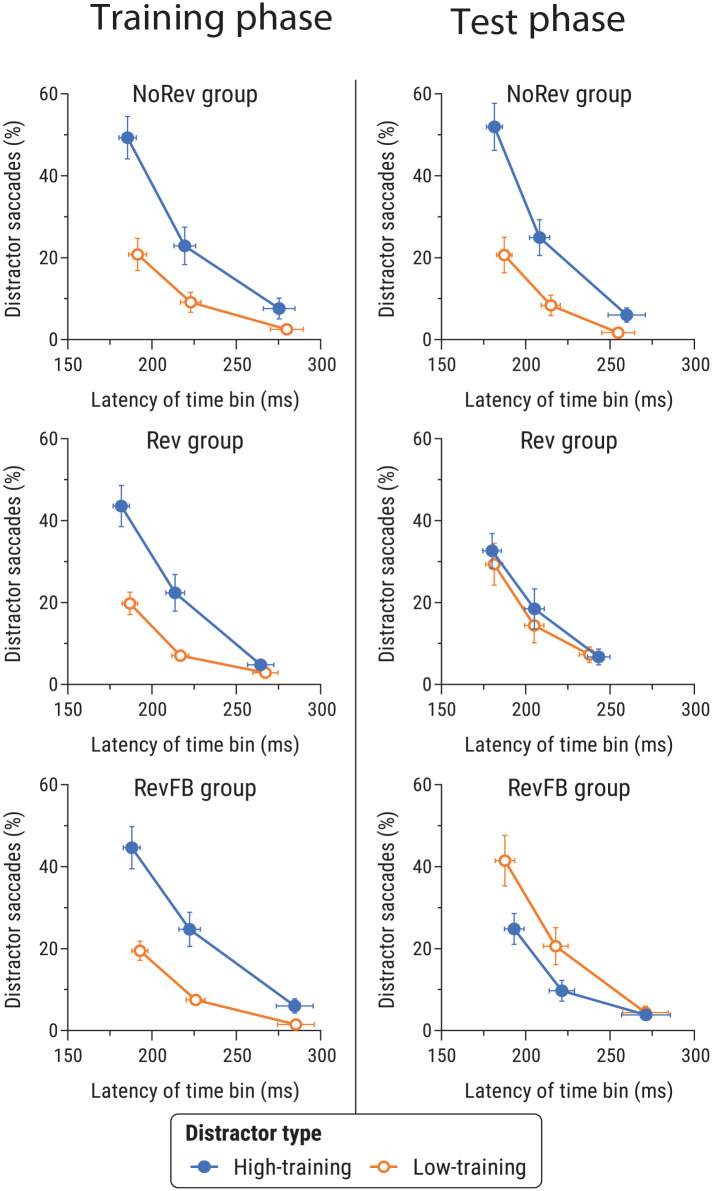
Proportion of saccades going towards the high- and low-training distractors as a function of mean saccade latency in the training phase (left) and the test phase (right) of Experiment 2, for the NoRev group (top), the Rev group (middle), and the RevFB group (bottom). See caption of Figure 3 for further details.

Figure 7 shows the proportion of first saccades that went towards distractors as a function of saccade latency. We analysed data for each phase using separate 2 (distractor-type: high- vs. low-training distractor) × 2 (revaluation group: NoRev, Rev, RevFB) × 3 (latency time bin: fastest, middle, slowest) ANOVAs.

##### Training phase

In the training phase, there was a significant main effect of distractor-type, *F*(1,71) = 49.82, *p* < .001, η_p_^2^ = .41 [.26, .52], with more first saccades towards the high-training distractor than the low-training distractor. There was also a significant main effect of saccade latency time bin, *F*(2,142) = 234.2, *p* < .001, η_p_^2^ = .77 [.71, .80], with shorter-latency saccades being more likely to go towards distractors. There was a significant interaction between these two factors, *F*(2,142) = 30.62, *p* < .001, η_p_^2^ = .30 [.19, .39], with the bias towards the high-training distractor over the low-training distractor being more pronounced at shorter saccade latencies. There were no significant effects involving revaluation group, *F*s < 0.31, *p*s > .87, η_p_^2^s < .009.

##### Test phase

In the test phase, ANOVA revealed a significant main effect of latency bin, *F*(2,136) = 154.2, *p* < .001, η_p_^2^ = .69 [.62, .74], with shorter-latency saccades more likely to go towards distractors. Notably a significant three-way interaction, *F*(4,136) = 7.17, *p* < .001, η_p_^2^ = .17 [.07, .25], indicated that the latency-modulated pattern of attentional bias differed across the three groups. There was also a significant distractor type × revaluation group interaction, *F*(2,68) = 9.00, *p* < .001, η_p_^2^ = .21 [.07, .33]. No other main effects or interactions were significant, *F*s < 1.79, *p*s > .185, η_p_^2^s < .03.

As for Experiment 1, follow-up analyses focused on data from the fastest time bin. Paired *t*-tests revealed a significant effect of distractor-type in the NoRev group, *t*(23) = 3.78, *p* < .001, *d_z_* = 0.77 [0.31, 1.22], with fastest saccades more often initiated towards the high-training distractor than the low-training distractor. In the Rev group, however, there was no significant effect of distractor type, *t*(23) = 0.49, *p* = .626, *d_z_* = 0.10 [-0.30, 0.50]; a Bayesian *t*-test assessing evidence for the one-tailed alternative hypothesis of greater rapid capture by the low-training distractor than the high-training distractor yielded *BF*_01_ = 6.51, representing moderate evidence in favour of the null hypothesis. Finally, in the RevFB group the fastest saccades were significantly more likely to go towards the low-training distractor than the high-training distractor, *t*(22) = 2.57, *p* = .017, *d_z_* = 0.54 [0.09, 0.97].

To test for between-group differences in attentional bias among the fastest saccades, we ran a series of 2 (revaluation group) ×2 (distractor-type) ANOVAs using the data from the fastest time bin. Contrasting the NoRev and Rev groups revealed a significant group × distractor-type interaction, *F*(1,46) = 6.93, *p* = .011, η_p_^2^ = .13 [.02, .28], with the NoRev group showing a greater rapid bias to high-training versus low-training distractors than the Rev group. Contrasting the Rev and RevFB groups again yielded a significant interaction, *F*(1,45) = 4.59, *p* = .038, η_p_^2^ = .09 [.003, .24], indicating that provision of outcome feedback in the test phase led to a difference in the resulting pattern of attentional bias following outcome revaluation. Finally, contrasting the RevFB and NoRev groups also revealed a significant interaction, *F*(1,45) = 20.59, *p* < .001, η_p_^2^ = .32 [.13, .46], in line with the reversal in the pattern of gaze bias in these groups observed in the data on overall proportion of distraction trials.

#### Knowledge checks

In each of the three groups, 26 participants reported the correct colour–fruit associations in the knowledge check at the end of the experiment. We repeated the above analyses of proportion of distraction trials while excluding the eight participants who failed to correctly identify the colour–fruit contingencies. These analyses did not affect the pattern of significant findings, except that—in the primary analysis of proportion of distraction trials—(1) the pattern of attentional bias in the test phase now differed significantly between Rev and NoRev groups, *F*(1,50) = 6.22, *p* = .016, η_p_^2^ = .11 [.01, .25], where previously this difference only came close to the threshold of significance (*p* = .051), and (2) the reversal of the attentional bias in the RevFB group (with more distraction trials for the low-training distractor than the high-training distractor) was now significant, *t*(25) = 2.22, *p* = .036, *d_z_* = 0.44 [0.03, 0.83], where previously it had only approached significance (*p* = .051).

### Discussion

Experiment 2 examined the effect of switching the values of outcomes on patterns of conditioned attentional bias. This instructed value-switch influenced the Rev group’s performance when tested in nominal extinction: this group no longer exhibited significantly greater attention to the high-training than the low-training distractor. Their bias towards the high-training distractor was also weaker than participants in the NoRev group, for whom outcome values were unchanged from training. This difference between NoRev and Rev groups was significant in focused analyses of participants’ fastest saccades, and in analyses of the proportion of distractor trials when we excluded participants who failed the explicit knowledge check regarding stimulus-outcome relationships.

Notably, we observed a significant difference in the pattern of attentional bias between the Rev and RevFB groups. Both underwent outcome revaluation, but the RevFB group continued to receive trial-by-trial feedback in the test phase, whereas the Rev group did not. Although participants in the Rev group had *knowledge* of the new values of the fruits, and knowledge of which fruit was signalled by each distractor, this knowledge alone was insufficient to overturn the attentional bias towards the high-training distractor in line with the updated outcome values. Only with the additional *experience* of the pairings of distractor colours and revalued outcomes—in the RevFB group—was evidence of a full reversal of attentional prioritisation observed. These participants showed a bias in overall proportion of distractor trials towards the low-training distractor that approached significance, and focused analysis of the fastest saccades showed a significant bias towards the low-training distractor. We consider these findings further in the “General discussion” section.

## General discussion

We investigated whether conditioned attentional prioritisation of reward-related stimuli was sensitive to acute changes in the values of outcomes. Interestingly, the degree to which attentional prioritisation of high- versus low-training distractors persisted following a change in outcome values varied across experiments. In Experiment 1, instructed revaluation following training rendered both fruits of equally low (Experiment 1a) or high (1b) value. In both cases, participants nevertheless continued to show an attentional bias towards the high-training distractor relative to the low-training distractor, regardless of whether or not trial-by-trial feedback was provided on the identity of the fruit earned during the test phase. By contrast, in Experiment 2 the values of the fruits were switched (high value became low value, and vice versa), and here we saw some evidence for a change in participants’ pattern of conditioned attentional prioritisation—with this change being most pronounced when participants received explicit outcome feedback during the test phase.

Taken together, our findings provide important insights into the influence of reward on attentional capture. Experiment 2 shows that a change in outcome value *can* result in updating of attentional priority, even when the stimulus–outcome (colour–fruit) relationship remains constant throughout; but Experiments 1a and 1b show that a change in value will not *always* lead to updating of priority. This in turn suggests that the persistence observed in Experiment 1 was a result of the procedure in which both outcomes had equal value following revaluation. These findings can be understood within Anderson’s (2021) “adaptive” view of attentional control, wherein updating of attentional priority is based on cost–benefit accounting derived from reinforcement learning. In Experiment 1, neither outcome was worth more than the other during test, so there may have been little incentive for participants to strive to earn a particular fruit, and hence little drive to exert (effortful) cognitive control to update attentional priority, with existing settings instead allowed to run on. Prior research indicates that a difference in *relative value*—as opposed to absolute value—is important for formation of value-modulated attentional biases (Kim & Beck, 2020); results of Experiment 2 suggest that a difference in relative value may also be critical for updating of existing biases. An implication of these findings is that revaluation procedures that equate outcome values (e.g., De Tommaso et al., 2017; De Tommaso & Turatto, 2021; Pool et al., 2014; Watson et al., 2022) may be suboptimal for detecting value-sensitivity of reward-modulated attention; instead procedures involving an ordinal change in outcome values may be better targeted.

Experiment 2’s finding that explicit knowledge of a reversal of outcome values was sufficient for partial updating of attentional priority suggests that the reward-related bias formed during training was (at least to some degree) mediated by a representation of the outcome and its current value, such that—under conditions providing motivation to update control settings—an instructed change in value produced a corresponding change in attention. However, the finding that additional experience of stimulus–outcome pairings produced further change in attention indicates that the bias formed in training was not entirely mediated by a representation of current outcome value. Instead direct experience seems to play an important role in updating. This implies that reward-related attentional biases, once formed, can become dissociated from explicit knowledge of the outcome’s current value, with subsequent retraining required for effective updating (cf. Berridge, 2012; Dickinson & Balleine, 1994).

In measuring the ability of reward-signalling stimuli to capture attention, our search task can be seen as a human analogue of *sign-tracking* in animals, wherein Pavlovian signals of reward gain incentive salience, becoming motivationally attractive and able to elicit appetitive behaviour in their own right (see Amaya et al., 2020; Berridge, 2012; Colaizzi et al., 2020). In a parallel with the current work, several animal studies have examined the influence of outcome revaluation on sign-tracking; findings have been mixed, with some work suggesting insensitivity (e.g., Morrison et al., 2015; Patitucci et al., 2016) and other studies finding that sign-tracking updates flexibly following a change in outcome value, suggesting mediation by a representation of outcome value (e.g., Derman et al., 2018; M. J. F. Robinson & Berridge, 2013). Recent research has argued that a key determinant of (in)flexibility in sign-tracking is the manner in which outcome revaluation is conducted, in terms of the congruence between the contexts in which revaluation and subsequent testing occur (Amaya et al., 2020). In highlighting the existence of external factors that determine whether effects of revaluation translate to modulate test-phase behaviour, this idea is broadly consistent with the implications of the current study. Indeed, our findings could also be seen as pointing to the importance of contextual congruence, in that revaluation was more effective when it produced a test phase in which there was a difference in relative outcome value (matching the situation in the training phase) than when it did not. This remains a question for future examination in studies of sign-tracking-like behaviour in humans. Given that sign-tracking in animals—and its analogue in humans—has been linked to the mechanisms underlying addiction and compulsive behaviour (e.g., Albertella et al., 2020; Albertella, Le Pelley, Chamberlain, et al., 2019; Colaizzi et al., 2020; Flagel et al., 2009; T. E. Robinson & Berridge, 2001), it is critical to understand when and how this behaviour might be rendered flexible versus rigid and “habit-like”—since this may point to factors influencing whether interventions will be successful or not.

Our study has some potential limitations. First, the use of the mediating fruit outcomes—while essential to our ability to isolate the influence of a change in outcome value from a change in outcome identity (see Introduction)—increased the complexity of the task. For outcome revaluation to influence patterns of established attentional bias, participants had to track and remember changes in outcome values of fruits, and then link those fruits to the respective distractor colours. However, it seems unlikely that this complexity is the reason for the null effect of outcome revaluation observed in Experiment 1. Notably, the procedure was just as complicated in Experiment 2—in fact it was more complicated, since revaluation involved changing the values of two outcomes concurrently rather than just one—and yet participants showed evidence of updating their attentional bias in Experiment 2. Thus we have evidence that updating *can* occur despite the complexity of this task, raising the question of why effects of revaluation were observed in Experiment 2 but not Experiment 1 (as outlined above).

A second limitation is that the critical reward-signalling distractors in this task were physically salient colour-singletons (as in our previous work with this task: e.g., Le Pelley et al., 2015; Pearson et al., 2015; Watson, Pearson, Chow, et al., 2019), and hence might be expected to capture attention to some degree on the basis of their physical salience (Theeuwes, 1992) regardless of their relationship with reward. Critically, the physical salience of high- and low-training distractors was matched across participants via counterbalanced assignment of specific colours to roles, such that at a group level these distractors differed only in their reward history. Consequently, our findings support previous research in demonstrating that reward can *change* the likelihood that distractors will capture attention, but could not assess whether reward can *cause* distractors to capture attention when they would not otherwise have done so. Future research could address this issue by using a variant of the procedure in which reward-signalling distractors are not physically salient (cf. Failing et al., 2015).

Finally, our sample sizes were somewhat limited, particularly for latency-based analyses which were subject to additional participant exclusions to preserve data quality, with some findings that were near the conventional threshold level of significance. Replication of these findings in larger samples would be valuable. This would also permit investigation of potential individual differences in flexibility of previously established patterns of reward-related attention, for example, in the context of addictive and compulsive behaviours (cf. Albertella, Le Pelley, Chamberlain, et al., 2019; Albertella, Watson, Yücel, et al., 2019; Liu et al., 2021).

In summary, we demonstrate that under some circumstances conditioned attentional prioritisation can have habit-like properties, persisting despite a change in outcome values. This has important implications for behavioural control, since attention plays a critical role in prioritising options for further consideration during decision making (see Gluth et al., 2018; Krajbich, 2019). While this prioritisation will be guided in part by our goals, demonstrations of reward-related attentional capture suggest that filtering will also be influenced by our previous experiences of outcome value (Pearson et al., 2022). Notably, the current findings suggest that this automatic prioritisation may persist even if values change so that outcomes are no longer as desirable. Our data suggest that such persistence would be most pronounced under conditions where there is little motivation to update existing attentional control settings (e.g., when there is no option available that is desired substantially more than others). In effect, this account proposes that attention can act to automatically prioritise certain possible goals and related courses of action. This process may play a role in maintaining maladaptive behaviours towards reward-related stimuli implicated in addiction and compulsive disorders despite attempts to abstain. More generally, the current findings provide a framework for future work investigating the relationship between attention and behavioural control.

## Supplemental Material

sj-pdf-1-qjp-10.1177_17470218241236711 – Supplemental material for Effects of outcome revaluation on attentional prioritisation of reward-related stimuli Supplemental material, sj-pdf-1-qjp-10.1177_17470218241236711 for Effects of outcome revaluation on attentional prioritisation of reward-related stimuli by Jenny T Le, Poppy Watson and Mike E Le Pelley in Quarterly Journal of Experimental Psychology

## Notes

1. In the current study, this would translate to the magnitude of the conditioned attentional bias dissipating over the course of “genuine” extinction. As noted earlier, existing evidence using a variant of the current procedure suggests that the magnitude of this bias may not diminish greatly over the course of a short extinction phase (Watson, Pearson, Most, et al., 2019), but we nevertheless thought it prudent to use nominal extinction to reduce the *potential* for a decrease, especially since the test phase of the current study was longer than in Watson et al.’s study.

2. The interval in square brackets gives a confidence interval (CI) on effect size; following convention we provide a 90% CI for values of η_p_^2^ and a 95% CI for Cohen’s *d*.

3. Prior studies of instrumental behaviour have often assessed the effectiveness of revaluation via *consumption tests*—participants see two outcomes (one still-valuable, the other devalued) and are asked which they would prefer to receive—with these consumption test trials interspersed throughout the test phase (e.g., Gillan et al., 2015; Luque et al., 2017). We instead used tests of explicit knowledge after the test phase, which has advantages: (1) it is a more conservative test of knowledge, requiring participants to directly report the value associated with each outcome; and (2) it lets us verify that this knowledge spanned the whole test phase.

4. Experiment 2 was actually run prior to Experiment 1, but we present the experiments out of chronological order for the sake of clear exposition—since the value-reversal manipulation of Experiment 2 effectively combines the devaluation and supervaluation manipulations of Experiments 1a and 1b.
