# Supplementary material for: Effects of outcome revaluation on attentional prioritisation of reward-related stimuli
Source: Q J Exp Psychol (Hove). 2024 Mar 15;78(1):142–62. doi: 10.1177/17470218241236711 (PMC11684138; doi:10.1177/17470218241236711)
Supplement: sj-pdf-1-qjp-10.1177_17470218241236711 – Supplemental material for Effects of outcome revaluation on attentional prioritisation of reward-related stimuli [file sj-pdf-1-qjp-10.1177_17470218241236711.pdf]

Supplementary Materials

to accompany

Effects of outcome revaluation on attentional prioritisation of reward-related stimuli

Jenny T. Le[1], Poppy Watson[1,2], & Mike E. Le Pelley[1]

[1] School of Psychology, UNSW Sydney, Sydney, Australia

[2] University of Technology, Sydney, Australia

Corresponding author:

Mike E. Le Pelley

School of Psychology, UNSW Sydney

Sydney NSW 2052

Australia

Email: m.lepelley@unsw.edu.au

This document has three sections:

1. Analysis of the proportion of distraction trials during the training phase as a function of training block (Training Analyses By Block).

2. Analysis of the proportion of distraction trials using data from the training phase restricted to only the last two blocks (Late-Training Analyses).

3. Record of numbers of retained participants and trials for latency-based analyses in each experiment (Retained Data in Latency-Based Analyses).

1. Training Phase Analyses By Block

In this section we report analyses of data from the training phase of each experiment as a function of training blocks, in order to examine how the attentional bias to the high-training distractor (versus the low-training distractor) developed over the course of training. In each experiment, the training phase comprised 16 blocks of trials, each containing 24 trials.

Experiment 1a

We examined the proportion of distraction trials across training using a 2 (distractor-type: high- vs. low-training distractor) × 16 (block) × 2 (group: Dev vs. DevFB) ANOVA. This revealed a significant main effect of distractor-type, $F(1,49) = 26.8$, $p < .001$, $\eta_p^2 = .35$ [.18, .49], with more distraction trials when the display contained a high-training distractor than a low-training distractor. There was also a significant distractor-type × block interaction, $F(15,735) = 4.74$, $p < .001$, $\eta_p^2 = .09$ [.04, .10], with the bias towards the high-training distractor increasing over the course of the training phase. No other main effects or interactions were significant. Figure S1a shows the proportion of distraction trials across blocks of the training phase of Experiment 1a; data have been collapsed over revaluation groups (Dev and DevFB) as these groups were treated identically until after the training phase (and the ANOVA found no effect of group).

Experiment 1b

Findings were similar to those of Experiment 1a: ANOVA revealed a significant main effect of distractor-type, $F(1,53) = 15.3$, $p < .001$, $\eta_p^2 = .22$ [.08, .37], and a significant distractor-type × block interaction, $F(15,795) = 3.23$, $p < .001$, $\eta_p^2 = .06$ [.02, .07], with the

bias towards the high-training distractor increasing over the course of the training phase (see Figure S1b).

Experiment 2

Findings were similar to those of previous experiments: ANOVA revealed a significant main effect of distractor-type, $F(1,82) = 50.8$, $p < .001$, $\eta_p^2 = .38$ [.25, .49], and a significant distractor-type × block interaction, $F(15,1230) = 6.63$, $p < .001$, $\eta_p^2 = .07$ [.04, .09], with the bias towards the high-training distractor increasing over the course of the training phase (see Figure S1c).

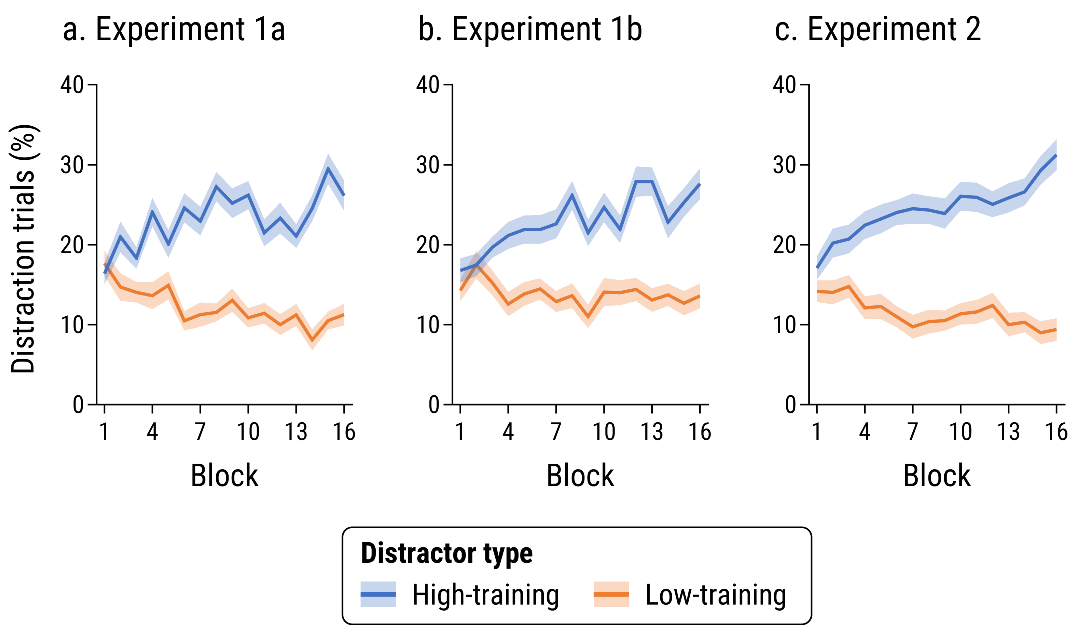

Figure S1. Proportion of distraction trials (i.e., trials in which participants looked at the coloured distractor) for trials featuring a high-training or low-training distractor, across blocks of trials in the training phase of (a) Experiment 1a, (b) Experiment 1b, and (c) Experiment 2. Data from each experiment are averaged across revaluation groups. Shaded region shows within-subjects *SEM* (Morey, 2008).

2. Test Phase Analyses By Block

In this section we report analyses of data from the test phase of each experiment as a function of training blocks, in order to examine whether and how patterns of attentional bias

changed over the course of the test phase training. In each experiment, the test phase comprised 8 blocks of trials, each containing 24 trials.

Experiment 1a

Figure S2a shows the proportion of distraction trials across blocks of the test phase of Experiment 1a. We examined these data using a 2 (distractor-type: high- vs. low-training distractor) × 8 (block) × 2 (group: Dev vs. DevFB) ANOVA. This revealed a significant main effect of distractor-type, $F(1,49) = 30.4$, $p < .001$, $\eta_p^2 = .38$ [.20, .52], with more distraction trials when the display contained a high-training distractor than a low-training distractor. Distractor-type did not interact with any other factors, largest $F(1,49) = 1.15$, $p = .290$, $\eta_p^2 = .023$ [0, .13]. The only other significant effect was the group × block interaction, $F(7,343) = 2.88$, $p < .006$, $\eta_p^2 = .056$ [.01, .08], suggesting that the pattern of performance across blocks (collapsed across distractor types) differed between groups – though there is not a clear and systematic pattern evident in Figure S2a. Importantly, this pattern did not interact with distractor-type, i.e, the pattern of reward-related attentional bias did not evolve differently across blocks in the different groups (and indeed, the lack of a distractor-type × block interaction suggests that the pattern of bias did not change significantly over the course of the test phase in general).

Experiment 1b

Data are shown in Figure S2b. Findings were similar to those of Experiment 1b: ANOVA revealed a significant main effect of distractor-type, $F(1,53) = 11.4$, $p = .001$, $\eta_p^2 = .18$ [.05, .32]. No other main effects or interactions reached significance, largest $F(1,53) = 1.84$, $p = .079$, $\eta_p^2 = .03$ [0, .14].

Experiment 2

Data are shown in Figure S2c. ANOVA with factors of distractor-type, block, and group (NoRev, Rev, RevFB) revealed a significant main effect of distractor-type, $F(1,83) = 5.20$, $p = .025$, $\eta_p^2 = .06$ [.004, .15], that was qualified by a significant interaction with group, $F(2,83) = 8.83$, $p < .001$, $\eta_p^2 = .18$ [.06, .28], consistent with the difference in the pattern of attentional bias between groups highlighted in analyses reported in the main text. No other main effects or interactions were significant, largest $F(14,581) = 1.56$, $p = .087$, $\eta_p^2 = .04$ [0, .04].

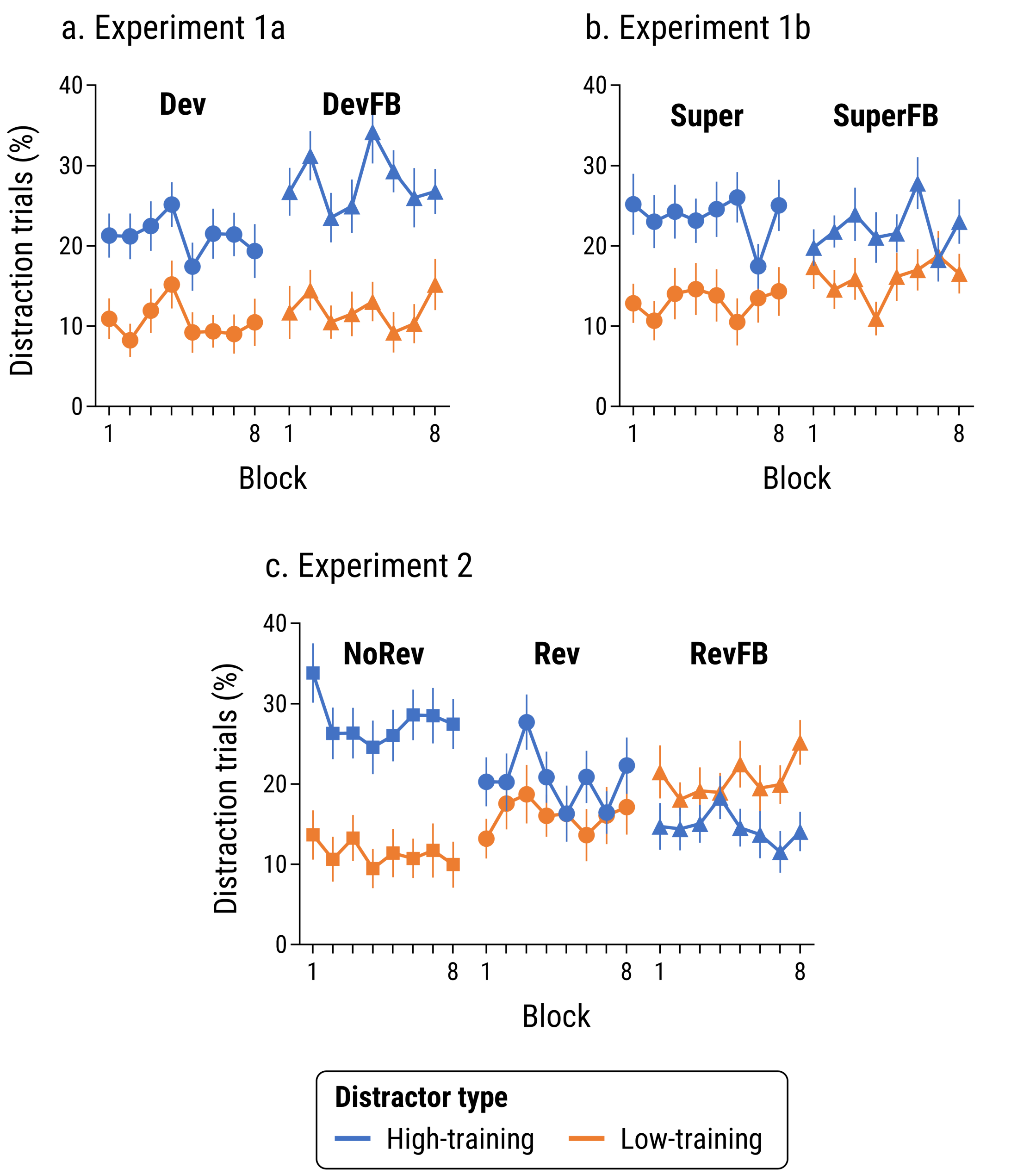

Figure S2. Proportion of distraction trials (i.e., trials in which participants looked at the coloured distractor) for trials featuring a high-training or low-training distractor, across blocks of trials in the test phase of (a) Experiment 1a, (b) Experiment 1b, and (c) Experiment 2. Error bars show within-subjects *SEM*.

3. Late-Training Analyses

In the analyses described in this section, data from the training phase of each experiment were restricted to only the final two trial-blocks prior the value switch (termed

the *late-training phase*), in order to examine the attentional bias immediately prior to the devaluation manipulation.

Experiment 1a

We examined the proportion of distraction trials across the late-training and test phases using a 2 (phase: late-training vs. test phase) × 2 (distractor-type: high- vs. low-training distractor) × 2 (group: Dev vs. DevFB) ANOVA. Notably, the three-way interaction was not significant, $F(1,49) = 0.001$, $p = .97$, $\eta_p^2 < .001$.

We then analysed data from the late-training phase in isolation, using ANOVA with factors of distractor-type and group. There was a significant main effect of distractor-type, $F(1,49) = 40.65$, $p < .001$, $\eta_p^2 = .45$ [.27, .57], with more distraction trials when the display contained a high-training distractor than a low-training distractor. There was no significant main effect of group, $F(1,49) = 2.03$, $p = .16$, $\eta_p^2 = .04$ [0, .16], or distractor-type × group interaction, $F(1,49) = 0.90$, $p = .35$, $\eta_p^2 = .02$ [0, .12].

Experiment 1b

We examined the proportion of distraction trials across the late-training and test phases using a 2 (phase: late-training vs. test phase) × 2 (distractor-type: high- vs. low-training distractor) × 2 (group: Super vs. SuperFB) ANOVA. Notably, the three-way interaction was not significant, $F(1,53) = 0.67$, $p = .42$, $\eta_p^2 = .01$ [0, .10].

We then analysed data from the late-training phase in isolation, using ANOVA with factors of distractor-type and group. There was a significant main effect of distractor-type, $F(1,53) = 16.84$, $p < .001$, $\eta_p^2 = .24$ [.09, .38], with more distraction trials when the display contained a high-training distractor than a low-training distractor. There was no significant

main effect of group, $F(1,53) = 0.23$, $p = .63$, $\eta_p^2 = .004$ [0, .07], or distractor-type × group interaction, $F(1,53) = 1.48$, $p = .23$, $\eta_p^2 = .03$ [0, .13].

Experiment 2

We examined the proportion of distraction trials across the late-training and test phases using a 2 (phase: late-training vs. test phase) × 2 (distractor-type: high- vs. low-training distractor) × 2 (group: NoRev, Rev, RevFB) ANOVA. Notably, the three-way interaction was significant, $F(2,83) = 6.68$, $p = .002$, $\eta_p^2 = .14$ [.03, .24], suggesting a significant difference in the distractor-type × group interaction between the training and test phases.

We then analysed data from the late-training phase in isolation, using a 2 (distractor-type: high- vs. low-training distractor) × 3 (revaluation group: NoRev, Rev, RevFB) ANOVA. There was a significant main effect of distractor-type, $F(1,83) = 75.36$, $p < .001$, $\eta_p^2 = .48$ [.34, .57], with a greater proportion of distraction trials when the display featured a high-training than a low-training distractor. There was no significant main effect of revaluation group, $F(2,83) = 0.23$, $p = .79$, $\eta_p^2 = .006$ [0, .04], nor was there a revaluation group × distractor-type interaction, $F(2,83) = 0.21$, $p = .81$, $\eta_p^2 = .005$ [0, .03]. These null findings are unsurprising, since all groups received equivalent treatment until after the training phase.

4. Retained Data in Latency-Based Analyses

Following previous work (Le Pelley et al., 2019; Pearson & Le Pelley, 2021; Pearson et al., 2016; Watson et al., 2020), for latency-based analyses (i.e., analysis of the direction of the first saccade on each trial as a function of its latency), trials were excluded if the start point of the saccade was not within 100 pixels of the central fixation point, if saccade latency was below 80 ms, if there were gaps in the gaze data which were too large to be interpolated, or

if there was insufficient gaze data to identify a saccade. Again in line with our standard protocols, any participants with more than 30% of invalid trials in a given phase of the task (training or test) were excluded from latency-based analyses of that phase. Table S1 shows numbers of retained participants and trials for each experiment.

Table S1. Retained participants and trials for latency-based analyses

			Training phase		Test phase	
Experiment	Group	Total *N*	Included participants	Valid trials	Included participants	Valid trials
Exp 1a	Dev	26	23	93.3%	22	95.1%
	DevFB	25	21	89.8%	16	91.8%
Exp 1b	Super	28	21	91.4%	24	91.4%
	SuperFB	27	19	84.9%	18	87.0%
Exp 2	NoRev	28	25	90.0%	24	93.1%
	Rev	29	23	91.6%	24	91.4%
	RevFB	29	26	90.4%	23	93.4%

Note: Participants were excluded from analysis of a given phase of an experiment if more than 30% of trials were invalid (if the start point of the saccade was not within 100 pixels of the central fixation point, if saccade latency was below 80 ms, if there were gaps in the gaze data which were too large to be interpolated, or if there was insufficient gaze data to identify a saccade). "Included participants" shows the number of participants from each group retained for analysis of each phase (training and test); "valid trials" shows the mean proportion of valid trials used in latency-based analysis for these subsamples of included participants.

References

Le Pelley, M.E., Pearson, D., Porter, A., Yee, H., & Luque, D. (2019). Oculomotor capture is influenced by expected reward value but (maybe) not predictiveness. *Quarterly Journal of Experimental Psychology, 72*, 168-181.

Morey, R.D. (2008). Confidence intervals from normalized data: A correction to Cousineau (2005). *Tutorial in Quantitative Methods for Psychology, 4*, 61-64.

Pearson, D., & Le Pelley, M.E. (2021). Reward encourages reactive, goal-directed suppression of attention. *Journal of Experimental Psychology: Human Perception and Performance, 47*, 1348-1364.

Pearson, D., Osborn, R., Whitford, T.J., Failing, M., Theeuwes, J., & Le Pelley, M.E. (2016). Value-modulated oculomotor capture by task-irrelevant stimuli is a consequence of early competition on the saccade map. *Attention, Perception, & Psychophysics, 78*, 2226-2240.

Watson, P., Pearson, D., & Le Pelley, M.E. (2020). Reduced attentional capture by reward following an acute dose of alcohol. *Psychopharmacology*.
